# Single-Trial Mechanisms Underlying Changes in Averaged P300 ERP Amplitude and Latency in Military Service Members After Combat Deployment

**DOI:** 10.3389/fnhum.2019.00377

**Published:** 2019-10-25

**Authors:** Amy Trongnetrpunya, Paul Rapp, Chao Wang, David Darmon, Michelle E. Costanzo, Dominic E. Nathan, Michael J. Roy, Christopher J. Cellucci, David Keyser

**Affiliations:** ^1^Henry M. Jackson Foundation, Department of Military and Emergency Medicine, Uniformed Services University, Bethesda, MD, United States; ^2^Department of Military and Emergency Medicine, Uniformed Services University, Bethesda, MD, United States; ^3^Department of Mathematics, Monmouth University, West Long Branch, NJ, United States; ^4^Department of Medicine, Uniformed Services University, Bethesda, MD, United States; ^5^Henry M. Jackson Foundation, Center for Neuroscience and Regenerative Medicine, Uniformed Services University, Bethesda, MD, United States; ^6^Graduate School of Nursing, Uniformed Services University, Bethesda, MD, United States; ^7^Aquinas LLC, Berwyn, PA, United States

**Keywords:** combat trauma, PTSD, ERP, P300, single-trial

## Abstract

Attenuation in P300 amplitude has been characterized in a wide range of neurological and psychiatric disorders such as dementia, schizophrenia, and posttraumatic stress disorder (PTSD). However, it is unclear whether the attenuation observed in the averaged event-related potential (ERP) is due to the reduction of neural resources available for cognitive processing, the decreased consistency of cognitive resource allocation, or the increased instability of cognitive processing speed. In this study, we investigated this problem by estimating single-trial P300 amplitude and latency using a modified Woody filter and examined the relation between amplitudes and latencies from the single-trial level to the averaged ERP level. ERPs were recorded from 30 military service members returning from combat deployment at two time points separated by 6 or 12 months. A conventional visual oddball task was used to elicit P300. We observed that the extent of changes in the within-subject average P300 amplitude over time was significantly correlated with the amount of change in three single-trial measures: (1) the latency variance of the single-trial P300 (*r* = −0.440, *p* = 0.0102); (2) the percentage of P300-absent trials (*r* = −0.488, *p* = 0.005); and (3) the consistent variation of the single-trial amplitude (*r* = 0.571, *p* = 0.0022). These findings suggest that there are multiple underlying mechanisms on the single-trial level that contribute to the changes in amplitudes seen at the averaged ERP level. The changes between the first and second assessments were quantified with the intraclass correlation coefficient, the standard error of measurement and the minimal detectable difference. The unique population, the small sample size and the large fraction of participants lost to follow up precludes generalizations of these measures of change to other populations.

## Introduction

The within-subject average P300 event-related potential (ERP) has demonstrated significant promise as an objective physiological measure of cognitive processing (Polich and Herbst, 2000). Variation in its amplitude (Pfefferbaum et al., 1989; Fabiani et al., 1990; Noldy et al., 1990; Polich, 1997), and latency have been well characterized in normal populations (McCarthy and Donchin, 1981; Verleger, 1997, 2010; Leuthold and Sommer, 1998; Doucet and Stelmack, 1999). Attenuation in P300 amplitude and slowing in P300 latency have also been associated with a wide range of neurological and psychiatric disorders such as dementia (Hedges et al., 2016), schizophrenia (Ford et al., 1994; Mathalon et al., 2000; Oribe et al., 2015), traumatic brain injury (Gaetz and Bernstein, 2001), and posttraumatic stress disorder (PTSD; McFarlane et al., 1993; Metzger et al., 1997; Kimble et al., 2000; Felmingham et al., 2002; Wang et al., 2017, 2018). The article by Ford et al. (1994) is particularly relevant to this contribution. Ford et al. (1994) examined P300s obtained from schizophrenics and from healthy comparison participants. They reported three observations. First, schizophrenics had fewer trials passing the P300 screen; that is, schizophrenics had fewer trials that elicited a response that satisfied the criterion for the presence of a P300. Second, the amplitude of P300s that passed criterion was smaller in schizophrenics, and, third, the single-trial latency was greater in schizophrenics. Demonstrating further potential for clinical use as a correlate to cognitive function, P300 has been shown to strengthen in response to various treatments (Kouri et al., 1996; Werber et al., 2003; Tilki et al., 2004; Chang et al., 2014; Khedr et al., 2014; Vaitkevičius et al., 2015). However, the mechanisms underlying the variations in within-subject average P300 remain unclear.

**Figure 1:**
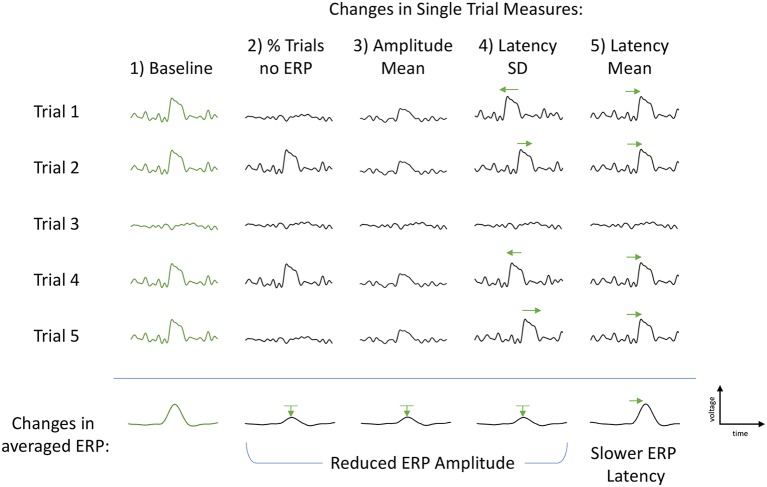
Illustration of the single-trial measures and their subsequent effects on the grand-averaged event-related potentials (ERPs). First column (Baseline, green): example of baseline trials with P300 elicited in four trials and absent in one trial. Second column, % Trls no ERP: each individual P300 remains unchanged, however the proportion of P300-absent trials increases. This is measured as a percent change in the number of P300-absent trials to total number of trials and results in a reduced classic grand-averaged P300 amplitude (bottom row). Third column, Amplitude Mean: each individual P300 is smaller in amplitude, number of P300-absent trials remains the same as baseline. Amplitude Mean is calculated as the mean of the estimated P300 amplitudes using only the four trials with an elicited P300. The middle P300-absent trial is not included in the calculation. The change in Amplitude Mean also results in a reduced classic grand-averaged P300 amplitude (bottom row). Fourth column, Latency Standard Deviation (SD): number of elicited P300s and their amplitudes are unchanged. Only variation is the latency of each P300 peak such that the latency variance (jitter) is increased (both slower and faster), but the average P300 latency remains constant, resulting in a smaller (and broader) averaged P300 amplitude (bottom row). Fifth column, Latency Mean: the latency of each P300 peak is consistently slower, resulting in a slower averaged ERP latency and no change to the ERP amplitude (bottom row). Green arrows used to help visually clarify shifts from baseline P300s (first column, green).

P300 is typically measured from an average of many single trials. Understanding the characteristics of the single trials may help to explain the differences in P300 seen in past studies and reveal insight into the underlying variation of cognitive processing. Changes in the amplitude of grand-averaged P300 ERPs may be due to a number of single-trial factors (Figure 1). First, the single-trial amplitudes may be smaller or larger across the trials that elicit a P300 ERP. Second, there may be a change in the proportion of trials that elicit a P300 ERP, and the proportion will be reflected in the within-subject average. Finally, an increase (decrease) in latency jitter could lead to smaller (larger) P300 ERP amplitude. Another single-trial effect that would not impact the change in within-subject average P300 ERP amplitude but would impact the change in ERP latency is a uniform shift in the single-trial latency mean.

Each of the above proposed alternative mechanisms has a different cognitive implication. Intermittently occurring P300s would be consistent with clinical reports of attentional fluctuations and distractibility in veterans with PTSD (Davis et al., 1996). Overall reduction of all P300-elicited trials would suggest the allocation of fewer resources for processing the stimulus, either due to depletion of available resources and/or to the misallocation of existing resources (Langer and Eickhoff, 2013). Single-trial latency variability could suggest fluctuations in processing strategy during data collection, change in instability or efficiency of cognitive processing, which would be consistent with studies relating single-trial latency variation to intra-individual reaction time characteristics (Saville et al., 2011) and of the association between aging and P300 attenuation (Daffner et al., 2006). The ability to extract meaningful information about alterations in neural resources available for cognitive processing within a single trial level of granularity could provide an objective metric of cognitive capacity. Disruption of neurocognitive function is a critical post-deployment health concern. it is influenced by factors that can be disrupted by combat exposure such as insomnia, stress and pain, making this of central importance when assessing deployment-related sequelae.

In this study, we aim to decouple these disparate underlying mechanisms by estimating the trial-by-trial P300 amplitude and latency using a modified Woody filter and examining the relations of amplitudes and latencies from the single-trial level to the averaged ERP level. Utilizing a longitudinal study design to monitor P300 ERP within-subject changes in the aftermath of combat trauma, we recruited military service members recently returned from a combat deployment in either Iraq or Afghanistan to undergo a baseline EEG assessment, with subsequent follow-up assessment at 6 or 12 months. The P300 ERPs were measured using a conventional visual oddball task paradigm.

## Materials and Methods

### Participants

Thirty military service members (age 30.4 ± 7.2 years, 27 men and three women) returned from a deployment of at least 3 months in either Iraq or Afghanistan were included in this study. Participants were not compensated for their participation. They completed a baseline ERP assessment within 2 months of their return, and a subsequent follow-up assessment at 6 or 12 months. Eighty-five candidate participants were screened. Of these 80 had a baseline EEG/ERP assessment. Of the 80, 10 had a follow-up EEG/ERP at 6 months and 28 had a follow-up at 12 months. The low retest rate is typical in studies of service personnel recently who returned from overseas duty. In many cases they are reassigned to other duty stations or separate from service. As will be addressed in a subsequent section of this article, the low retest rate has significant implications for the interpretation of quantitative measures of retest reliability. At the time of baseline assessment, participants did not meet the diagnostic criteria for PTSD, major depressive disorder or post-concussion syndrome. Exclusion criteria were the following: a current Glasgow Coma Scale score less than 13; a history of head injury resulting in loss of consciousness for 60 min or more; visual acuity lower than 20/100 after correction; psychosis; active suicidal or homicidal ideation; pregnancy; a PTSD Checklist-Military Version score (Forbes et al., 2001) greater than or equal to 50, or a diagnosis of PTSD made by an experienced psychologist using the Clinician-Administered PTSD Scale (Weathers et al., 2001) based on the DSM-IV criteria; a diagnosis of post-concussion syndrome according to the International Classification of Diseases, 10th Clinical Modification; and a Patient Health Questionnaire-9 score (Spitzer et al., 1999 and the Patient Health Questionnaire Primary Care Study Group) greater than or equal to 10.

All participants provided written informed consent in accordance with the protocol approved by institutional review boards at Uniformed Services University, Walter Reed National Military Medical Center, and the National Institutes of Health.

### Task Paradigm

Scalp EEG was recorded at both baseline and follow-up assessments while participants performed a visual oddball task. Visual stimuli were presented by a digital tachistoscope of our own design and construction. The tachistoscope was a 5 × 5 square array of yellow, light-emitting diodes. Each diode was 1 cm in diameter. Given spacing between LEDs, the array was 6 × 6 cm. The standard visual stimulus was a vertical stimulus which consists of the five vertical center line LEDs illuminated simultaneously for 40 ms. The target visual stimulus was a horizontal stimulus which was composed of the five horizontal center line LEDs illuminated simultaneously for 40 ms. Each subject received 125 stimuli in total, of which about 21% (26 ± 1 trials) were target and 79% (99 ± 1 trials) were standard stimuli. The subjects were instructed to maintain a silent count of the number of target stimulus presentations and to report their count at the end. The inter-stimulus onset time was varied randomly between 1.4 and 1.8 s. The experiment lasted about 3.5 min.

Recordings were obtained in a steel-enclosed electromagnetically shielded chamber that was lined with sound absorbent material. Gold electrodes were used and the impedance of each channel was less than 5 KΩ. Low level ambient light was on throughout the procedure. Prior to initiation of the task, participants were instructed from a standardized script. The task was described and the participant was asked to respond “as quickly and as accurately as possible.” The recording was preceded by practice trials to ensure that the participant understood the task.

### EEG Recording

The scalp EEG was recorded using an EPA6 amplifier (Sensorium Inc.) and Grass electrodes (Natus Neurology Inc.) at Fz, Cz, Pz, Oz, C3, and C4 according to the standard 10–20 electrode system, with linked earlobes as reference and a forehead ground. Electrode impedances were maintained under 5 KΩ. EOG was recorded from two electrodes placed above and below the right eye. The sampling rate was 2,048 Hz, and the analog filter band-pass was 0.02–500 Hz.

### EEG Data Processing

EEG data were analyzed offline using custom scripts written in MATLAB^1^. The continuous EEG signals from each participant were first visually inspected. Channels with poor signal quality were removed from further analysis. EOG artifacts were corrected by using a regression approach (Croft and Barry, 1998). After EOG correction the data were high-pass filtered at 1 Hz and low-pass filtered at 50 Hz. Continuous EEG data were then segmented into epochs from −500 to 1,000 ms with respect to the stimulus onset. Trials with activity exceeding ±75 μV were excluded from analysis. The overall trial rejection rate was 4.2%. The rejection rates for target and standard stimuli were 4.6% and 4.1%. The ERP waveforms for target and standard stimuli were extracted by averaging those preprocessed epochs. Electrode location of maximum P300 activation, Pz, was used for all further analysis. For each subject, the averaged P300 ERP amplitude and latency were measured as the voltage of the largest positive peak of target ERP within 250–500 ms and the time from stimulus onset to the maximum positive amplitude within 250–500 ms, respectively.

### Single-Trial P300 Analysis

Analysis was limited to responses to target stimuli. Single-trial latencies and amplitudes were determined by calculating the correlation between a single trial and a template that was determined using the procedure presented in Thornton et al. (2007) and Thornton (2008). An iterated procedure is used to produce the template.

1. The average of all single trials, T, is computed.
2. Single trials are divided into three subgroups, A, B and C corresponding to the first, second and third of trials in recorded order.
3. The average of each subgroup is calculated.
4. The lag between T and the Subgroup A is determined and denoted by L_A_. Similarly, the lag between T and the average of Subgroup B is L_B_ and the lag between T and the average of Subgroup C is L_C_.
5. A new template is formed by averaging Subgroup A single trials shifted by L_A_ with Subgroup B single trials shifted by L_B_ with Subgroup C trials shifted by L_C_.
6. The process is re-entered at Step 4 using the new template.
7. The process continues to iterate until the difference between the iterated templates is less than a prespecified difference. Thornton et al. (2007) use the phrase “until no further changes” result.

The number of subgroups is then increased by three and the process continues with trials divided across six subgroups until, as before, the iterated template is stable. The increase in subgroup number continues until the number of subgroups is equal to or just below one half the total number of single trials. By using this procedure all shift latencies used to calculate the template are determined from correlations determined between average signals (the current template correlated with the average of a subgroup). This prevents the possibility, present in latencies determined between a template and a single trial, of a maximum correlation lag obtained with a signal component that is due to noise.

The three resulting outputs determined by the maximum correlation with the template were: (1) an estimated peak P300 latency for each trial and (2) the corresponding P300 amplitude at that peak latency for each trial, along with (3) a correlation coefficient per trial which indicated how close each trial matched with the averaged P300 ERP template. Subsequently, trials were defined as having an elicited ERP if they had a correlation coefficient greater than 0.3. The legitimacy of this criterion was investigated by reviewing a very large number of single trials visually to determine if ERPs would be inappropriately lost when this criterion was used. We did not observe instances where this occurred with the 0.3 correlation criterion. Of note, this threshold was varied from 0.1 to 0.4, with no change on the significance of the results. Trials without an ERP were removed and tracked as single-trial-level measure in and of itself, as “% P300-absent trials.” The remaining trials were then used to calculate the mean and standard deviation (SD) of the single-trial amplitudes and single-trial latencies.

The following values were obtained from each participant using within-subject average ERPs:

1. Baseline amplitudes of the within-subject average ERP
2. Follow-up amplitudes of the within-subject averaged ERP
3. The difference of these amplitudes (Follow-up-Baseline)
4. Baseline latencies of the within-subject average ERP
5. Follow-up latencies of the within-subject average ERP
6. The difference of these latencies (Follow-up-Baseline)

The following values are obtained from each participant using the distributions determined from the participant’s set of single trials:

1. Baseline percentage of trials with no ERP
2. Baseline distribution of single-trial amplitudes (mean and standard deviation)
3. Baseline distribution of single-trial latencies (mean and standard deviation)
4. Follow-up percentage of trials with no ERP
5. Follow-up distribution of single-trial amplitudes (mean and standard deviation)
6. Follow-up distribution of single-trial latencies (mean and standard deviation)
7. Change in single-trial mean amplitudes (Follow-up-Baseline)
8. Change in single-trial latencies (Follow-up-Baseline)
9. Change in the percentage of trials with no ERP (Follow-up-Baseline)

Pearson correlations are calculated between changes in statistics characterizing the average ERP and changes in statistics that characterize single-trial distributions (Table 1). The *P*-values and confidence intervals for the correlation coefficients were determined using the percentile bootstrap with 10,000 bootstrap samples per measure. Confidence intervals are adjusted using the Bonferroni correction to have overall coverage probability of 95% within each table. Each table lists 10 comparisons, so this corresponds to a 99.5% coverage probability for each individual confidence interval. The *p*-values reported are not adjusted, and if the reader wishes to compare them to the common 0.05 and 0.01 significance level, they should use the adjusted significance levels of 0.005 and 0.001, respectively. Note that using a Bonferroni correction will generally lead to a conservative (lower than prescribed) family-wise error rate when test statistics are correlated, as should be expected for the correlations amongst the pre-post measures. This results in reduced power to detect true effects. However, as with any hypothesis test, there is always a tradeoff between the ability to detect true effects while avoiding the detection of spurious effects, and we have chosen to err on the side of avoiding the detection of spurious effects.

**Table 1:** Pearson correlations between changes in parameters characterizing the within-subject average event-related potential (ERP) and changes in the distributions of single-trial measures.

	Change in amplitude of within-subject average ERP	Change in latency of within-subject average ERP
Change in mean latency of single trials	*r* = −0.16 (−0.59, 0.40) *p* = 0.42	*r* = 0.62 (0.027, 0.89) *p* = 0.01
Change in the SD of latencies of single trials	*r* = −0.44 (−0.79, 0.047) *p* = 0.01	*r* = 0.12 (−0.29, 0.49) *p* = 0.38
Change in mean amplitude of single trials	*r* = 0.57 (0.046, 0.90) *p* = 0.01	*r* = −0.28 (−0.64, 0.28) *p* = 0.11
Change in SD of amplitudes of single trials	*r* = 0.037 (−0.47, 0.57) *p* = 0.83	*r* = −0.026 (−0.48, 0.35) *p* = 0.82
Change in % trials with no ERPs	*r* = −0.49 (−0.78, −0.0013) *p* = 0.01	*r* = 0.23 (−0.45, 0.73) *p* = 0.47

*The estimated correlation (confidence interval) for each pair is reported, as well as the bootstrapped *p*-value. Confidence intervals have been adjusted for 10 multiple comparisons using the Bonferroni correction to have simultaneous confidence level of 95%*.

## Results

**Figure 2:**
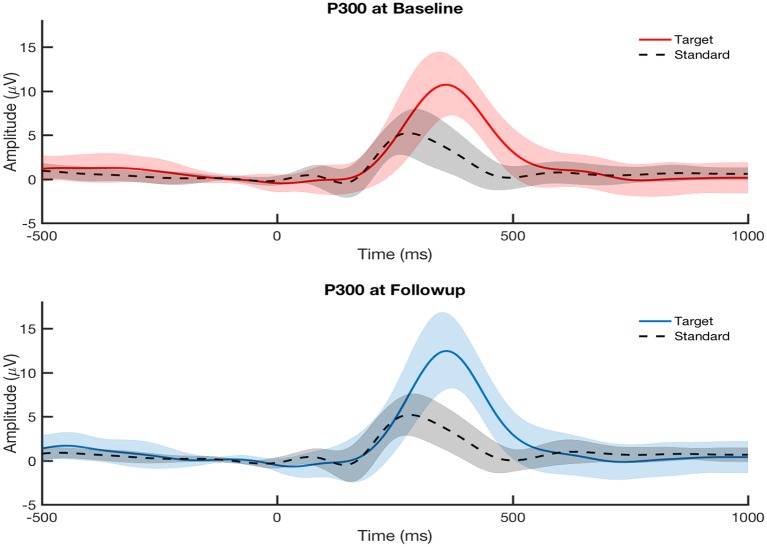
Grand-average visually-evoked P300 ERPs from central parietal channel (Pz) at baseline visit (top, red) and follow-up visit (bottom, blue) across all participants (*N* = 30) for both attended (target) and ignored (standard) conditions. Follow-up assessment was taken at either 6 months or 12 months after baseline assessment. Shaded areas indicate the standard error of the mean. P300 ERPs showed no significant difference between baseline and follow-up visits at the group level.

The P300 amplitude and latency were measured from each participant’s ERP at electrode Pz. Figure 2 shows that the participants overall did not show any differences in their averaged P300 from baseline to follow-up assessment at the group level. This is expected since the cohort was studied longitudinally with no clinical diagnoses for PTSD, major depressive disorder, or post-concussion syndrome at baseline and no specified treatment between baseline and follow-up. We examined the within-subject correlations between the changes (calculated as follow-up minus baseline) in P300 measures on the grand-averaged level and single-trial level (Table 1).

**Figure 3:**
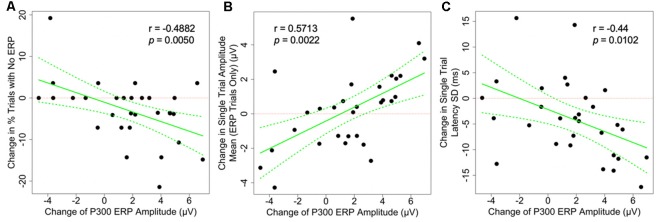
Scatter plots showing the correlation between the changes of grand-averaged P300 ERP amplitude and **(A)** the change in the proportion of P300-absent trials (% trials with no ERP, not the number of trials), **(B)** the change in single-trial P300 amplitude mean, and **(C)** the change in latency SD. All correlations are statistically significant, suggesting that there are multiple mechanisms underlying the changes seen on the averaged ERP level. Dotted green lines indicate a 95% confidence band for the regression curve.

As shown in Figure 3, the changes in the average P300 ERP amplitude are significantly correlated to two of the hypothesized underlying single-trial measures as illustrated in Figure 1, column 2–4 and plotted in Figure 3. First, P300 amplitude was negatively correlated with percentage of P300-absent trials out of the total number of trials (*r* = −0.488, *p* = 0.005). Second, P300 amplitude was positively correlated with amplitude mean (*r* = 0.571, *p* = 0.0022). The P300 amplitude was not significantly correlated with latency SD (*r* = −0.44, *p* = 0.0102) after correction for multiple comparisons, however, its confidence interval (−0.795, 0.047) is suggestive of anything from a moderately negative correlation to a very weak positive correlation. The observed correlations amongst the remaining single-trial measures, the change in single-trial P300 latency mean and amplitude SD, and the change in averaged P300 ERP amplitude did not achieve statistical significance at the 0.05 significance level after adjusting for multiple comparisons (Table 1).

**Figure 4:**
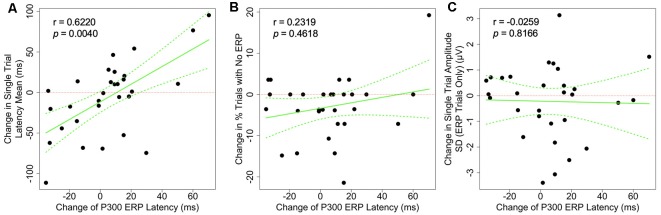
Scatter plots showing the correlation between the changes of grand-averaged P300 ERP latency and **(A)** the change single-trial latency mean, **(B)** the change in the proportion of P300-absent trials (% trials with no ERP), and **(C)** the change in amplitude SD. Dotted green lines indicate a 95% confidence band for the regression curve.

The associations between the change in P300 ERP latency and the change in each of the single-trial measures are shown in Figure 4. We found that the changes in P300 average ERP latency was positively correlated with the changes in the P300 latency mean on a single-trial level (*r* = 0.622, *p* = 0.004). This result is consistent with the expected electrophysiological effect of the P300 latency mean on the classic averaged P300 latency (Figure 1, last column). No other observed correlations achieved the 0.05 significance level after adjustment for multiple comparisons (Table 1).

### Quantifying T1 to T2 Differences

This is not a clinical population. As indicated in the description of the inclusion/exclusion criteria, participants were excluded based on traumatic brain injury history, suicidal ideation, PTSD symptoms, psychological symptoms identified by the Patient Health Questionnaire and the presence of persistent post-concussion symptoms. The measures quantifying single-trial ERPs reported in this article cannot, therefore, be correlated with major clinical pathologies, as was done in, for example, Ford et al. (1994). These measures indicate, admittedly provisionally given the small sample size, the test-retest reliability of these measures as quantified by the intraclass correlation coefficient, the ICC. There are several variants of the ICC. Shrout and Fleiss (1979) published six and McGraw and Wong (1996) published 10. Guidelines for selecting the appropriate version are given in Müller and Büttner (1994). Following that guidance, we report here ICC_(2,1)_ for five measures (Table 2).

**Table 2:** Quantifying T1 to T2 Differences.

Measure	ICC_(2,1)_	Standard error of measurement	Minimal detectable difference
Amplitude of the intrasubject P300	0.807 (0.636, 0.903)	1.496 (1.061, 2.055)	4.148 (2.941, 5.697)
Intrasubject single-trial P300 amplitude variance	0.564 (0.265, 0.765)	0.992 (0.728, 1.288)	2.750 (2.019, 3.571)
Latency of the intrasubject average P300	0.535 (0.226, 0.747)	31.859 (23.500, 41.104)	88.310 (65.139, 113.934)
Intrasubject single-trial P300 latency variance	0.408 (0.072, 0.664)	5.817 (4.382, 7.283)	16.124 (12.148, 20.188)
Frequency of rejected trials	0.358 (0.028, 0.625)	0.055 (0.042, 0.067)	0.152 (0.116, 0.187)

*Five measures were assessed twice. The T1 to T2 test-retest reliability is quantified using the ICC_(2,1)_ intraclass correlation coefficient. These coefficients and the corresponding standard deviations are used to calculate the Standard Error of Measurement and the Minimal Detectable Difference*.

The implications of these results for clinical practice must be considered with caution. Because the intraclass correlation coefficient can be used to calculate the clinically important Standard Error of Measurement and the Minimal Detectable Difference (Portney and Watkins, 2015, also reported for these measures in the table), it might be thought possible to use these measures longitudinally to assess treatment response or disease progression. This requires interpretation of intraclass correlation coefficients. To a limited degree, a comparative sense of the intraclass correlation coefficients obtained here can be obtained by comparing them to ICCs obtained with psychophysiological measures known to be stable in a healthy population, for example, measures of heart rate variability computed from RR interval sequences (the sequence of time intervals separating peaks of successive QRS complexes in the ECG). Killian et al. (2015) report the following from healthy adult controls at rest: mean RR interval (ICC_(2,1)_ = 0.791), SD of RR intervals (ICC_(2,1)_ = 0.831), root mean square of successive RR intervals (ICC_(2,1)_ = 0.814) and ratio low frequency to high frequency bands of the RR spectrum (ICC_(2,1)_ = 0.886). ICC’s obtained with the ERP data analyzed here are discernibly lower. Portney and Watkins provide the following general guidance (Portney and Watkins, 2015, pp. 594–595): “As a general guideline, we suggest that values above 0.75 are indicative of good reliability, and those below 0.75 poor to moderate reliability. For many clinical measurements, reliability should exceed 0.90 to ensure reasonable validity. These are only guidelines, however, and should not be used as absolute standards. Researchers and clinicians must defend their judgments within the context of the specific scores being assessed and the degree of acceptable precision in the measurement” (emphasis in the original text).

Additional considerations further temper the possibility of clinical utility. In addition to the limitations of the sample size in this study, it must be noted that reliability coefficients are population-dependent. A change that may be clinically significant in an age-matched civilian population, which typically displays highest reliability, may well be at noise level in a population of returned service personnel who have experienced combat exposure and some of whom may on entry into the study be below clinical threshold but prodromal for significant psychopathology. These intraclass correlation coefficients do not, therefore, generalize to other populations. The high loss to follow-up (the failure to obtain a second assessment) is an additional cause of concern. As previously noted, this low retest rate is typical in studies of service personnel recently returned from overseas duty (which is the population of interest to this program). This is a problem in reliability studies because it cannot be assumed that the measures under investigation are uniformly distributed across the groups that did and did not receive a second assessment. The population available for a second assessment may be significantly different from the population lost to follow-up.

In aggregate, these considerations argue that at least in this population the measures of ERPs examined here will have limited longitudinal clinical utility when used in isolation as single measures. It remains possible that a continuation study with a larger population and possibly using additional measures of ERP dynamics may be more successful. Also, greater utility might be obtained when these measures are combined with other data. in a multivariate assessment of change.

## Discussion

We utilized a longitudinal, repeated-measures study design to investigate the underlying mechanisms for the variation seen in grand-averaged ERPs. Within 2 months after their return from combat deployment, 30 military service members were asked to perform a visual oddball P300 assessment as a baseline measure, then again after 6 or 12 months. Since classically averaged ERPs are computed as an average over multiple trials with any trial-to-trial variation averaged out, we examined the P300 measures on a single-trial level in order to understand better the variations seen in the averaged P300 measures. We observed that the variation in P300 amplitude was significantly associated with changes in single-trial amplitude mean and the proportion of P300-absent trials. Similarly, P300 latency was significantly associated with the changes in single trial latency mean. These results altogether were consistent with the hypothesized single-trial measures contributing to the changes in the averaged ERP shown in Figure 1 and provide evidence of multiple electrophysiological mechanisms underlying the variation in averaged ERP amplitude.

P300 is thought to be a non-specific measure of cognitive health, reflective of fundamental cognitive processes including attention allocation of cortical resources, memory storage, and processing efficiency (Polich, 2007). The P300 component has been widely studied in normal populations (Polich and Herbst, 2000; Verleger et al., 2005) and implicated in a wide range of neurological disorders ranging from cognitive decline with aging, to depression, PTSD, autism, schizophrenia, TBI, and Alzheimers (Oken, 1997; Pan et al., 1999; Reinvang, 1999; Cycowicz, 2000; Jeon and Polich, 2003; Verleger, 2003; Polich, 2004; DeBoer et al., 2004). As such, many studies have decomposed the P300 component into single-trial measures. However, the methodological approach to single-trial analyses has varied. Many studies treated some of these single-trial measures such as latency jitter as task-irrelevant variation to be corrected in order to calculate true P300 amplitude (Roth et al., 1980; Walhovd et al., 2008). Other studies focused solely on intra-subject variation, both to prove its neurophysiological significance and its relation to normal and pathological measures (Ritter et al., 1972; Kutas et al., 1977; Blankertz et al., 2011; Biscaldi et al., 2016; Ouyang et al., 2017). Here we propose that the seemingly conflicting results or partial results all fit into a unified approach considering the multiplicity of P300 mechanisms behind the variations in the classic grand-averaged P300, and that examining the single-trial measures in addition to the averaged P300 would be a more informative step toward decoupling the different possible mechanisms.

Abnormalities in P300 has been implicated in a wide range of neurological disorders, including delayed-onset PTSD, depression, and neuropsychological and cognitive deficits due to mTBI (Kimble et al., 2000; Karl et al., 2006; Javanbakht et al., 2011; Johnson et al., 2013; Proudfit et al., 2015), all of which are risk factors for our specific cohort of combat-exposed yet currently clinically healthy veterans. Again attention is directed to the essential work of Ford et al. (1994) who found that schizophrenics present increased latency jitter, an increased fraction of trials that do not elicit a P300 and smaller single-trial ERP amplitudes. Many studies have tried to link these deficits and disorders with potential underlying causes, such as structural, vascular, etc. Glushakova et al. (2014) have shown evidence of evolving white matter degeneration following TBI, associated with microvascular abnormalities leading to blood-brain barrier damage and progressive inflammatory responses (Araki et al., 2005; Glushakova et al., 2014; Taib et al., 2017). Each of these injuries would have different implications on the electrophysiological impact. Reduction in P300 amplitude may be associated with a multitude of abnormalities, including reduced volume of the anterior cingulate cortex gray matter (Araki et al., 2005) and reduced white matter integrity (Fjell et al., 2011; Tamnes et al., 2012), all of which could contribute to increasing P300 latency delay and variability. Loss of dopamine D1 receptors in caudate and DLPFC (MacDonald et al., 2012) reported in a longitudinal study in Parkinson patients found shortened P300 latency significantly related to dopaminergic systems. Future studies are needed to determine the relations between the individual single-trial measures and their possible structural and physiological causes.

Several limitations of this study should be considered. First, this was a group that began the study with no clinically diagnosed pathologies. Moreover, the P300 latency and amplitude can be influenced by several internal and external factors such as exercise (Yagi et al., 1999), fatigue (Haubert et al., 2018), age and gender (Polich and Herbst, 2000; Ribeiro and Castelo-Bianco, 2019) Taking into consideration within-subject ERP variability between visits, especially months apart, in addition to the baseline normalcy of our cohort, the participants ideally should be more accurately separated into three groups (deteriorated, stable, and improved) instead of two groups (deteriorated and improved). Last, our linear correlation results may ignore nonlinearity from an individual’s cognitive capacity to compensate for injury (Wang et al., 2018). Future studies with greater sample size are needed to properly explore the contributions of each single-trial measure to the strengthening or weakening of the average P300 ERP.

In conclusion, we propose that single-trial analysis may, therefore, serve as a valuable approach to assess cognitive processing and mental health. We demonstrated evidence of multiple electrophysiological mechanisms underlying the variation in averaged ERP amplitude. Here, we propose a unified approach of multiple P300 mechanisms influencing the variations in the classic grand-averaged P300, and that examining the single-trial measures in addition to the averaged P300 could be a step towards decoupling the different possible mechanisms.

## Data Availability Statement

The datasets for this manuscript are held by the United States Department of Defense and are not publicly available. Requests to access the datasets should be directed to Paul Rapp (paul.rapp@usuhs.edu).

## Ethics Statement

The studies involving human participants were reviewed and approved by Institutional Review Board, Uniformed Services University of the Health Sciences in accordance with all applicable Federal regulations governing the protection of research participants. The patients/participants provided their written informed consent to participate in this study.

## Author Contributions

AT acquired the data, conducted the primary analysis and wrote the first draft of the article. PR designed the study and participated in the analysis of the data and revision of the article. CW participated in the acquisition of the data and its analysis. MC established inclusion/exclusion of the participants and conducted the psychological evaluation of participants. DD conducted the statistical analysis and participated in the design of the study. DN participated in data acquisition. MR participated in the design of the study. CC designed and maintained the data acquisition system. DK directed laboratory operations, participated in data acquisition and in the drafting of the manuscript.

## Conflict of Interest

CC was employed by Aquinas, LLC. The remaining authors declare that the research was conducted in the absence of any commercial or financial relationships that could be construed as a potential conflict of interest.

## Footnotes

^1^www.mathworks.com

## References

[B1] Araki T. Kasai K. Yamasue H. Kato N. Kudo N. Ohtani T.. (2005). Association between lower P300 amplitude and smaller anterior cingulate cortex volume in patients with posttraumatic stress disorder: a study of victims of Tokyo subway sarin attack. Neuroimage 25, 43–50. 10.1016/j.neuroimage.2004.11.039 15734342

[B3] Biscaldi M. Bednorz N. Weissbrodt K. Saville C. W. N. Feige B. Bender S.. (2016). Cognitive endophenotypes of attention deficit/hyperactivity disorder and intra-subject variability in patients with autism spectrum disorder. Biol. Psychol. 118, 25–34. 10.1016/j.biopsycho.2016.04.064 27143193

[B4] Blankertz B. Lemm S. Treder M. Haufe S. Müller K. R. (2011). Single-trial analysis and classification of ERP components—a tutorial. Neuroimage 56, 814–825. 10.1016/j.neuroimage.2010.06.048 20600976

[B5] Chang Y.-S. Chen H.-L. Hsu C.-Y. Tang S.-H. Liu C.-K. (2014). Parallel improvement of cognitive functions and P300 latency following Donepezil treatment in patients with Alzheimer’s disease: a case-control study. J. Clin. Neurophysiol. 31, 81–85. 10.1097/01.wnp.0000436899.48243.5e 24492450

[B6] Croft R. J. Barry R. J. (1998). EOG correction: a new perspective. Electroencephalogr. Clin. Neurophysiol. 107, 387–394. 10.1016/s0013-4694(98)00086-8 9922083

[B7] Cycowicz Y. M. (2000). Memory development and event-related brain potentials in children. Biol. Psychol. 54, 145–174. 10.1016/s0301-0511(00)00055-7 11035222

[B8] Daffner K. R. Ryan K. K. Williams D. M. Budson A. E. Rentz D. M. Wolk D. A.. (2006). Increased responsiveness to novelty is associated with successful cognitive aging. J. Cogn. Neurosci. 18, 1759–1773. 10.1162/jocn.2006.18.10.1759 17014379

[B9] Davis J. M. Adams H. E. Uddo M. Vasterling J. J. Sutker P. B. (1996). Physiological arousal and attention in veterans with posttraumatic stress disorder. J. Psychopathol. Behav. Assess. 18, 1–20. 10.1007/bf02229099

[B10] DeBoer T. Scott L. Nelson C. A. (2004). “Event-related potentials in developmental populations,” in Event Related Potentials. A Methods Handbook, ed. Handy T. (Cambridge, MA: MIT Press), 263–297.

[B11] Doucet C. Stelmack R. M. (1999). The effect of response execution on P3 latency, reaction time and movement time. Psychophysiology 36, 351–363. 10.1017/s0048577299980563 10352559

[B12] Fabiani M. Karis D. Donchin E. (1990). Effects of mnemonic strategy manipulation in a Von Restorff paradigm. Electroencephalogr. Clin. Neurophysiol. 75, 22–35. 10.1016/0013-4694(90)90149-e 1688770

[B13] Felmingham K. L. Bryant R. A. Kendall C. Gordon E. (2002). Event-related potential dysfunction in posttraumatic stress disorder: the role of numbing. Psychiatry Res. 109, 171–179. 10.1016/s0165-1781(02)00003-3 11927142

[B14] Fjell A. M. Westlye L. T. Amlien I. K. Walhovd K. B. (2011). Reduced white matter integrity is related to cognitive instability. J. Neurosci. 31, 18060–18072. 10.1523/JNEUROSCI.4735-11.2011 22159119 PMC6634144

[B15] Forbes D. Creamer M. Biddle D. (2001). The validity of the PTSD checklist as a measure of symptomatic change in combat-related PTSD. Behav. Res. Ther. 39, 977–986. 10.1016/s0005-7967(00)00084-x 11480838

[B16] Ford J. M. White P. Lim K. O. Pfefferbaum A. (1994). Schizophrenics have fewer and smaller P300s: a single-trial analysis. Biol. Psychiatry 35, 96–103. 10.1016/0006-3223(94)91198-3 8167215

[B17] Gaetz M. Bernstein D. M. (2001). The current status of electrophysiologic procedures for the assessment of mild traumatic brain injury. J. Head Trauma Rehabil. 16, 386–405. 10.1097/00001199-200108000-00008 11461660

[B18] Glushakova O. Y. Johnson D. Hayes R. L. (2014). Delayed increases in microvascular pathology after experimental traumatic brain injury are associated with prolonged inflammation, blood-brain barrier disruption, and progressive white matter damage. J. Neurotrauma 31, 1180–1193. 10.1089/neu.2013.3080 24564198

[B19] Haubert A. Walsh M. Boyd R. L. Morris M. Wiedbusch M. Krusmark M.. (2018). Relationship of event-related potentials to the vigilance decrement. Front. Psychol. 9:237. 10.3389/fpsyg.2018.00237 29559936 PMC5845631

[B20] Hedges D. Janis R. Mickelson S. Keith C. Bennett D. Brown B. L. (2016). P300 amplitude in Alzheimer’s disease: a meta-analysis and meta-regression. Clin. EEG Neurosci. 47, 48–55. 10.1177/1550059414550567 25253434

[B21] Javanbakht A. Liberzon I. Amirsardri A. Gjini K. Boutros N. N. (2011). Event-related potential studies of post-traumatic stress disorder: a critical review and synthesis. Biol. Mood Anxiety Disord. 1:5. 10.1186/2045-5380-1-5 22738160 PMC3377169

[B22] Jeon Y.-W. Polich J. (2003). Meta-analysis of P300 and schizophrenia: patients, paradigms, and practical implications. Psychophysiology 40, 684–701. 10.1111/1469-8986.00070 14696723

[B23] Johnson J. D. Allana T. N. Medlin M. D. Harris E. W. Karl A. (2013). Meta-analytic review of P3 components in posttrauamtic stress disorder and clinical utility. Clin. EEG Neurosci. 44, 112–134. 10.1177/1550059412469742 23545246

[B24] Karl A. Malta L. S. Maercker A. (2006). Meta-analytic review of event-related potential studies in post-traumatic stress disorder. Biol. Psychol. 71, 123–147. 10.1016/j.biopsycho.2005.03.004 15961210

[B26] Khedr E. M. Gamal N. F. E. El-Fetoh N. A. Khalifa H. Ahmed E. M. Ali A. M. (2014). A double-blind randomized clinical trial on the efficacy of cortical direct current stimulation for the treatment of Alzheimer’s disease. Front. Aging Neurosci. 6:275. 10.3389/fnagi.2014.00275 25346688 PMC4191219

[B27] Killian J. M. Radin R. M. Gardner C. L. Kauske L. Bashirelahi K. Nathan D. (2015). Low Cost Alternative Devices for Heart Rate Variability Measures. A Test-Retest Reliability Study. Technical Report, Department of Military and Emergency Medicine, Uniformed Services University.

[B28] Kimble M. Kaloupek D. Kaufman M. Deldin P. (2000). Stimulus novelty differentially affects attentional allocation in PTSD. Biol. Psychiatry 47, 880–890. 10.1016/s0006-3223(99)00258-9 10807961

[B29] Kouri E. M. Lukas S. E. Mendelson J. H. (1996). P300 assessment of opiate and cocaine users: effects of detoxification and buprenorphine treatment. Biol. Psychiatry 40, 617–628. 10.1016/0006-3223(95)00468-8 8886295

[B30] Kutas M. McCarthy G. Donchin E. (1977). Augmenting mental chronometry: the P300 as a measure of stimulus evaluations. Science 197, 792–795. 10.1126/science.887923 887923

[B31] Langer R. Eickhoff S. B. (2013). Sustaining attention to simple tasks: a meta-analytic review of neural mechanisms of vigilant attention. Psychol. Bull. 139, 870–900. 10.1037/a0030694 23163491 PMC3627747

[B32] Leuthold H. Sommer W. (1998). Postperceptual effects and P300 latency. Psychophysiology 35, 34–46. 10.1017/s0048577298960553 9499704

[B33] MacDonald S. W. Karlsson S. Rieckmann A. Nyberg L. Bäckman L. (2012). Aging-related increases in behavioral variability: relations to losses of dopamine D1 receptors. J. Neurosci. 32, 8186–8191. 10.1523/JNEUROSCI.5474-11.2012 22699899 PMC6703653

[B34] Mathalon D. H. Ford J. M. Pfefferbaum A. (2000). Trait and state aspects of p300 amplitude reduction in schizophrenia: a retrospective longitudinal study. Biol. Psychiatry 47, 434–449. 10.1016/s0006-3223(99)00277-2 10704955

[B35] McCarthy G. Donchin E. (1981). A metric for thought: a comparison of P300 latency and reaction time. Science 211, 77–80. 10.1126/science.7444452 7444452

[B36] McFarlane A. C. Weber D. L. Clark C. R. (1993). Abnormal stimulus processing in posttraumatic stress disorder. Biol. Psychiatry 34, 311–320. 10.1016/0006-3223(93)90088-u 8399831

[B37] McGraw K. O. Wong S. P. (1996). Forming inferences about some intraclass correlation coefficients. Psychol. Methods 1, 30–46. 10.1037/1082-989x.1.1.30

[B38] Metzger L. J. Orr S. P. Lasko N. B. Pitman R. K. (1997). Auditory event-related potentials to tone stimuli in combat-related posttraumatic stress disorder. Biol. Psychiatry 42, 1006–1015. 10.1016/s0006-3223(97)00138-8 9386852

[B39] Müller R. Büttner P. (1994). A critical discussion of intraclass correlation coefficients. Stat. Med. 13, 2465–2476. 10.1002/sim.4780132310 7701147

[B40] Noldy N. E. Stelmack R. M. Campbell K. B. (1990). Event-related potentials and recognition memory for pictures and words: the effects of intentional and incidental learning. Psychophysiology 27, 417–428. 10.1111/j.1469-8986.1990.tb02337.x 2236443

[B41] Oken B. S. (1997). “Endogenous event-related potentials,” in Evoked Potentials in Clinical medicine, eds Chiappa K. K. (Philadelphia, PA: Lippincott-Raven), 529–564.

[B43] Oribe N. Hirano Y. Kanba S. Del Re E. Seidman L. Mesholam-Gately R.. (2015). Progressive reduction of visual P300 amplitude in patients With first-episode schizophrenia: an ERP Study. Schizophr. Bull. 41, 460–470. 10.1093/schbul/sbu083 24914176 PMC4332938

[B44] Ouyang G. Hildebrandt A. Sommer W. Zhou C. (2017). Exploiting the intra-subject latency variability from single trial event related potentials in the P3 time range: a review and comparative evaluation of methods. Neurosci. Biobehav. Rev. 75, 1–21. 10.1016/j.neubiorev.2017.01.023 28137458

[B45] Pan J.-B. Takeshita T. Morimoto K. (1999). P300 as a measure of cognitive dysfunction from occupational and environmental insults. Environ. Health Prev. Med. 4, 103–110. 10.1007/bf02932264 21432181 PMC2723518

[B46] Pfefferbaum A. Ford J. M. White P. M. Roth W. T. (1989). P3 in schizophrenia is affected by stimulus modality, response requirements, medication status, and negative symptoms. Arch. Gen. Psychiatry 46, 1035–1044. 10.1001/archpsyc.1989.01810110077011 2573328

[B47] Polich J. (1997). On the relationship between EEG and P300: individual differences, aging, and ultradian rhythms. Int. J. Psychophysiol. 26, 299–317. 10.1016/s0167-8760(97)00772-1 9203011

[B50] Polich J. (2004). Clinical application of the P300 event-related brain potential. Phys. Med. Rehabil. Clin. N. Am. 15, 133–161. 10.1016/s1047-9651(03)00109-8 15029903

[B51] Polich J. (2007). Updating P300: an integrative theory of P3a and P3b. Clin. Neurophysiol. 118, 2128–2148. 10.1016/j.clinph.2007.04.019 17573239 PMC2715154

[B48] Polich J. Herbst K. L. (2000). P300 as a clinical assay: rationale, evaluation, and findings. Int. J. Psychophysiol. 38, 3–19. 10.1016/s0167-8760(00)00127-6 11027791

[B52] Portney L. G. Watkins M. P. (2015). Foundations of Clinical Research. Applications to Practice. 3rd Edn. Philadelphia, PA: F. A. Davis Company.

[B53] Proudfit G. H. Bress J. N. Foti D. Kujawa A. Klein D. N. (2015). Depression and event-related potentials: emotional disengagement and award insensitivity. Curr. Opin. Psychol. 4, 110–113. 10.1016/j.copsyc.2014.12.018 26462292 PMC4598954

[B54] Reinvang I. (1999). Cognitive event-related potentials in neuropsychological assessment. Neuropsychol. Rev. 9, 231–248. 10.1023/A:1021638723486 10667449

[B55] Ribeiro M. Castelo-Bianco M. (2019). Age-related differences in event-related potentials and pupillary responses in cued reaction time tasks. Neurobiol. Aging 73, 177–189. 10.1016/j.neurobiolaging.2018.09.028 30366291

[B56] Ritter W. Simson R. Vaughan H. G. (1972). Association cortex potentials and reaction time in aduitory discrimination. Electroencephalogr. Clin. Neurophysiol. 33, 547–555. 10.1016/0013-4694(72)90245-3 4117332

[B57] Roth W. R. Horvath T. B. Pfefferbaum A. Kopell B. S. (1980). Event-related potentials in schizophrenics. Electroencephalogr. Clin. Neurophysiol. 48, 127–139. 10.1016/0013-4694(80)90299-0 6153330

[B58] Saville C. W. N. Dean R. O. Daleyu D. Intriligator J. Boehm S. Feibe B.. (2011). Electrocortical correlates of intra-subject variability in reaction times: average and single trial analyses. Biol. Psychol. 87, 74–83. 10.1016/j.biopsycho.2011.02.005 21335053

[B59] Shrout P. E. Fleiss J. L. (1979). Intraclass correlations: uses in assessing rater reliability. Psychol. Bull. 86, 420–428. 10.1037/0033-2909.86.2.420 18839484

[B60] Spitzer R. L. Kroenke K. Williams J. W. The Patient Health Questionnaire Primary Care Study Group. (1999). Validation and utility of a self-report version of prime-md: the PHQ primary care study. JAMA 282, 1737–1744. 10.1001/jama.282.18.1737 10568646

[B62] Taib T. Leconte C. Van Steenwinckel J. Cho A. H. Palmier B. Torsello E. (2017). Neuroinflammation, myelin and behavior: temporal patterns following mild traumatic brain injury in mice. PLoS One 12:e0184811. 10.1371/journal.pone.0184811 28910378 PMC5599047

[B63] Tamnes C. K. Fjell A. M. Westlye L. E. Østby Y. Walhovd K. B. (2012). Becoming consistent: developmental reductions in intraindividual variability in reaction time are related to white matter integrity. J. Neurosci. 32, 972–982. 10.1523/jneurosci.4779-11.2012 22262895 PMC6621149

[B64] Thornton A. J. (2008). Evaluation of a technique to measure latency jitter in event related potentials. J. Neurosci. Methods 168, 248–255. 10.1016/j.jneumeth.2007.09.031 18006068

[B200] Thornton A. R. D. Harmer M. Lavoie B. A. (2007). Selective attention increases the temporal precision of the auditory N100 event-related potential. Hearing Res. 230, 73–79. 10.1016/j.heares.2007.04.004 17606341

[B65] Tilki H. E. Akpolat T. Tunalı G. Kara A. Onar M. K. (2004). Effects of haemodialysis and continuous ambulatory peritoneal dialysis on P300 cognitive potentials in uraemic patients. Ups. J. Med. Sci. 109, 43–48. 10.3109/2000-1967-109 15124952

[B66] Vaitkevičius A. Kaubrys G. Audronytė E. (2015). Distinctive effect of Donepezil treatment on P300 and N200 subcomponents of auditory event-related evoked potentials in Alzheimer disease patients. Med. Sci. Monit. 21, 1920–1927. 10.12659/msm.894940 26138001 PMC4501636

[B67] Verleger R. (1997). On the utility of P3 latency as an index of mental chronometry. Psychophysiology 34, 131–156. 10.1111/j.1469-8986.1997.tb02125.x 9090263

[B68] Verleger R. (2003). “Event related potential research in neurological patients,” in The Cognitive Psychophysiology of Mind and Brain, eds Zani A. Proverbio A. M. (Amsterdam: Academic Press), 309–341.

[B69] Verleger R. (2010). Popper and P300: can the view ever be falsified tjhat P3 latency is a specific indicator of stimulus evaluation? Clin. Neurophysiol. 121, 1371–1372. 10.1016/j.clinph.2010.01.038 20363185

[B70] Verleger R. Jaśkowski P. Wascher E. (2005). Evidence for an integrative role of P3b in linking reactions to perception. J. Psychophysiol. 19, 165–181. 10.1027/0269-8803.19.3.165

[B71] Walhovd K. B. Rosquist H. Fjell A. M. (2008). P300 amplitude age reductions are not caused by latency jitter. Psychophysiology 45, 545–553. 10.1111/j.1469-8986.2008.00661.x 18346042

[B72] Wang C. Costanzo M. E. Rapp P. E. Darmon D. Bashirelahi K. Nathan D. E.. (2017). Identifying electrophysiological prodromes of post-traumatic stress disorder: results from a pilot study. Front. Psychiatry 8:71. 10.3389/fpsyt.2017.00071 28555113 PMC5430065

[B73] Wang C. Rapp P. Darmon D. Trongnetrpunya A. Costanzo M. Nathan D.. (2018). Utility of P300 ERP in monitoring post-trauma mental health: a longitudinal study in military personnel returning from combat deployment. J. Psychiatr. Res. 101, 5–13. 10.1016/j.jpsychires.2018.02.027 29522937

[B75] Weathers F. W. Keane T. M. Davidson J. R. (2001). Clinician-administered PTSD scale: a review of the first ten years of research. Depress. Anxiety 13, 132–156. 10.1002/da.1029 11387733

[B76] Werber E. A. Gandelman-Marton R. Klein C. Rabey J. M. (2003). The clinical use of P300 event related potentials for the evaluation of cholinesterase inhibitors treatment in demented patients. J. Neural Transm. 110, 659–669. 10.1007/s00702-003-0817-9 12768361

[B78] Yagi Y. Coburn K. L. Estes K. M. Arruda J. E. (1999). Effects of aerobic exercise and gender on visual and auditory P300, reaction time, and accuracy. Eur. J. Appl. Physiol. Occup. Physiol. 80, 402–408. 10.1007/s004210050611 10502073

